# Non-surgical treatment of canine oral malignant melanoma: A case study of the application of complementary alternative medicine

**DOI:** 10.3892/ol.2014.2041

**Published:** 2014-04-07

**Authors:** HIROYASU ITOH, TOSHIYUKI MUKAIYAMA, TAKAHIRO GOTO, KEISHI HATA, KAZUO AZUMA, TAKASHI TSUKA, TOMOHIRO OSAKI, TOMOHIRO IMAGAWA, YOSHIHARU OKAMOTO

**Affiliations:** 1 Kamo Animal Clinic, Saijyocho, Higashihiroshima, Hiroshima 739-0044, Japan; 2 Sakamoto Bio Co. Ltd., Yuuwa-Memeki, Akita 010-1233, Japan; 3 Institute of Food and Brewing, Akita Prefectural Agricultural, Forestry and Fisheries Research Center, Arayamachi, Akita 010-1623, Japan; 4 Faculty of Agriculture, Tottori University, Koyama-Minami, Tottori 680-8553, Japan

**Keywords:** canine oral malignant melanoma, complementary alternative medicine, quality of life, hyperthermia, lupeol

## Abstract

This report describes a dog with a clinical stage III oral malignant melanoma that was treated with complementary alternative medicine (CAM). The CAM included high temperature hyperthermia, dendritic cell therapy and lupeol injections. Surgery, radiation and chemotherapy were not performed. Two months after the start of treatment, the tumor disappeared and after six months, the follow-up examinations revealed no recurrence or metastasis of the tumor. Quality of life (QOL) of the dog was maintained; therefore, the application of CAM may be an effective treatment for canine oral malignant melanoma. The effective application of CAM has the potential to prolong life and maintain an excellent QOL for pets.

## Introduction

In the field of veterinary medicine, canine melanoma is the most common type of malignant oral cancer (1). Previous studies have reported a median survival time of 219–273 days in cases of canine oral malignant melanoma, which were treated with surgery (2–4), with only 164 days in cases of clinical stage II or III canine oral malignant melanoma that were treated with surgery (5). Chemotherapy and radiation have been reported to be unable to prolong the survival of dogs with oral malignant melanoma (6,7). The present report describes a dog with stage III oral malignant melanoma, which was treated via non-surgical complementary alternative medicine (CAM). The dogs owner provided written informed consent and the study was approved by the ethics committee of Kamo Animal Clinic (Saijyocho, Higashihiroshima, Japan).

## Case report

**Figure 1:**
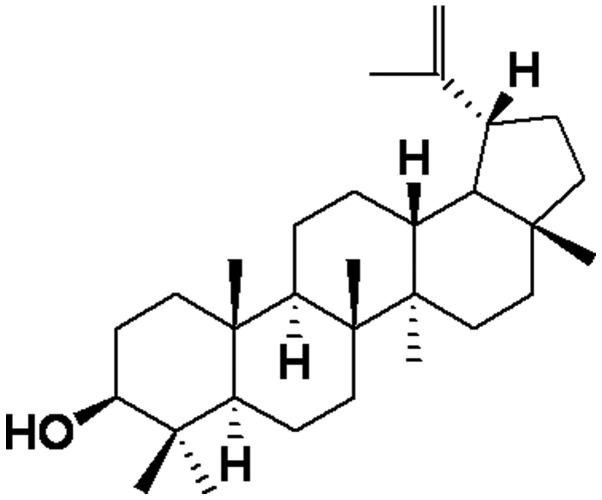
Chemical structure of lupeol.

A 12-year-old male Labrador retriever (weight, 39 kg) was diagnosed with malignant melanoma of the left jaw (day 0). The diameter of the tumor was ~4.0 cm and no metastasis to the lung was observed via radiography. The dog was diagnosed with a clinical stage III oral malignant melanoma in accordance with World Health Organization guidelines (8). With consent from the owner, application of CAM, including high temperature hyperthermia, injections of lupeol (Fig. 1), dendritic cell therapy and oral administration of extracts of *Cordyceps sinensis* (Monolis Inc., Saitama, Japan) were performed.

Our previous study demonstrated that lupeol, a lupane triterpene, induced the differentiation of human and mouse melanoma cells. Lupeol induced melanogenesis and suppressed proliferation and mobility of melanoma cells *in vitro* (9). Thus, in the present study, 5 mg/ml lupeol was dissolved in olive oil and this solution following heat sterilization at 150°C for 90 min was used in the differentiation-inducing therapy for the dog. On day seven, 5 and 10 ml lupeol solution was injected topically and subcutaneously, respectively, and the original site was treated using a high temperature hyperthermia device (AMTC 200, AdMeTech Co., Ltd., Ehime, Japan). The hyperthermia device was inserted into the primary site and heated at 65°C for 8 min. In addition, melanoma cells (>5.0 mm in diameter) were collected from the original site and used as antigen-presenting cells for the dendritic cells. The dendritic cell culture was performed in accordance with the manufacturer’s instructions (J-ARM Inc., Osaka, Japan). Subcutaneous injections of 15 ml lupeol were administered on days 11, 14 and 21. As recurrence of the melanoma was detected on day 24, the recurrence site was treated using the same hyperthermia device (AMTC 200). Cultured dendritic cells (1.1×10^6^) and 2.5 ng/kg interleukin (IL)-12 (J-ARM Inc.) were injected into numerous sites surrounding the melanoma and the precordial region of the lower jaw, respectively. Lupeol (15 ml) was subcutaneously injected on days 34 and 47. As the melanoma recurred on day 54, it was treated using the same hyperthermia device (AMTC 200) and 5 and 15 ml lupeol was injected topically and subcutaneously, respectively. In addition, 15 ml lupeol was subcutaneously injected once every two weeks and an X-ray on day 142 revealed no metastasis. On day 211, second injections of dendritic cells (1.2×10^6^) and IL-12 were administered as mentioned above. Currently, the dog maintains a good quality of life (QOL) without recurrence or metastasis.

## Discussion

Previous studies have reported that chemotherapy and radiation are unable to prolong the survival time of dogs with oral malignant melanoma (6,7). Novel alternative treatments for cancer using an experimental model have been investigated. One such treatment is the injection of lupeol, which is a lupane triterpene that is contained in fruits, such as olives, mangos, strawberries, grapes, figs, as well as several vegetables and medicinal plants (10). Our previous study demonstrated that systemic and local injections of lupeol suppressed tumor growth and induced cell cycle arrest in a melanoma-bearing mouse model (11). Furthermore, high-temperature hyperthermia has been shown to suppress tumor growth (12). In conclusion, the application of CAM in the present study prolonged the life of a dog with oral malignant melanoma and, therefore, may be considered to be as effective as surgical and radiation therapy. Furthermore, an excellent QOL was maintained. Continued investigation into effective treatments is important to establish additional non-surgical treatment methods for canine oral malignant melanoma.
